# Controlled Atmosphere Brazing of 3003 Aluminum Alloy Using Low-Melting-Point Filler Metal Fabricated by Melt-Spinning Technology

**DOI:** 10.3390/ma15176080

**Published:** 2022-09-01

**Authors:** Zeng Gao, Zhen Qin, Qingsong Lu

**Affiliations:** 1 School of Materials Science and Engineering, Henan Polytechnic University, Jiaozuo 454003, China; 2 Zhejiang Yinlun Machinery Co., Ltd., Taizhou 317200, China; 3 Key Laboratory of Smart Thermal Management Science & Technology for Vehicles of Zhejiang Province, Taizhou 317200, China

**Keywords:** aluminum alloy, low melting point, filler metal, melt spinning, controlled atmosphere brazing

## Abstract

3003 aluminum alloy was widely used for the manufacturing of heat exchangers in the automotive industry by employing controlled atmosphere brazing (CAB) with NOCOLOK flux brazing technology. However, commercially available filler metals for NOCOLOK flux brazing technology are usually required to be carried out at a relatively high temperature, causing the assembled heat exchanger to be partially molten or easily deformed. A new low-melting-point brazing filler metal Al-5.0Si-20.5Cu-2.0Ni was prepared by using melt-spinning technology and then applied to CAB of 3003 aluminum alloy in this research. The solidus and liquidus of brazing filler metal was 513.21 °C and 532.48 °C. All elements were evenly distributed and free from elemental segregation. The microstructure of brazing filler metal was uniform, and the grain size was less than 500 nm. As the brazing temperature reached 575 °C, the void in the joint disappeared completely. The morphology of CuAl_2_ was sensitive to the brazing temperature and dwell time. The appearance of net-like CuAl_2_ brazed at 575 °C for 20 min was more beneficial to improve joint mechanical properties. The leakage rate of the joint was qualified to be 10^−10^ Pa·m^3^/s when the brazing temperature was 570 °C or higher. The maximum shear strength of 76.1 MPa can be obtained when the joint was brazed at 575 °C for 20 min. More dwell time induced growth of the interfacial layer and reduced joint shear strength. The open circuit potential and corrosion current density test indicated that the brazing filler metal Al-5.0Si-20.5Cu-2.0Ni had better corrosion resistance than that of 3003 aluminum alloy.

## 1. Introduction

In past decades, aluminum alloys are widely used for the manufacturing of heat exchangers in the automotive industry instead of copper alloys due to their comparative advantages, such as low cost, low density, satisfactory mechanical properties, relatively good thermal conductivity, and good corrosion resistance, etc. [1,2,3]. In industrial production, 3003 aluminum alloy plays a major role in the manufacturing of heat exchangers, where controlled atmosphere brazing (CAB) with non-corrosive fluxes (NOCOLOK flux brazing technology) is the most widely used process characterized by high efficiency and low cost [4,5]. Commercially available filler metals for NOCOLOK flux brazing technology are usually targeted at Al-12.6Si alloy, which has a eutectic temperature at about 577 °C. However, brazing with that conventional filler metal requires the process to be carried out at a relatively high temperature, generally above 600 °C [6]. As a result, the assembled heat exchanger made of 3003 aluminum alloy may be partially molten or deformed in an industrial furnace during the brazing process, even causing brazing failure. In addition to this, an excessive brazing temperature not only increases the fabricating cost, but also causes dissolution of the base material [7]. It is desirable to develop a low-melting-point filler metal to satisfy the brazing of 3003 aluminum alloy used for the heat exchanger industry.

In the past few years, many related research studies have been conducted to develop low-melting-point filler metal for brazing of aluminum alloys. Based on the eutectic components of Al-Si, Al-Si-Cu, Al-Zn, Al-Mg, Al-Ge, and so on, several alloy systems have been developed to solve the problem of an excessively high brazing temperature with the assistance of the alloying method [8,9,10,11]. Al-Si alloy is the most widely used brazing filler metal in aluminum heat exchanger production due to its good wettability and forming performance. The brazing joint also has high mechanical properties and good corrosion resistance. However, the high brazing temperature may cause grain growth of the base metal or even over-burning [12,13]. Al-Si-Cu filler metal has good wettability and a low melting point. However, many researchers found that there were many θ (CuAl_2_) brittle phases in the joint, making for a lower shear strength. Moreover, the existence of a large number of θ brittle phases makes it difficult for the brazing filler metal to be formed into thin sheets through the traditional rolling method [14,15,16]. The liquidus temperature of Al-Zn alloy is from 380 °C to 450 °C. Although it has a perfect melting temperature, its wettability is much worse than that of Al-Si alloy, and more importantly, the corrosion resistance of Al-Zn alloy is much lower than that of Al-Si alloy, which makes it difficult to be used in heat exchangers [17,18]. Mg can break oxide film on the surface of aluminum alloys in vacuum conditions since Mg has stronger atomic activity and higher vapor pressure than Al, making Al-Mg alloy often used as a vacuum brazing filler metal [19,20,21]. However, due to the low production efficiency of vacuum brazing technology and the pollution of Mg steam to equipment, its application is not as wide as that of NOCOLOK flux brazing technology. In addition to this, it is not feasible to apply Al-Mg filler metal to NOCOLOK flux brazing technology because Mg can significantly destroy NOCOLOK flux. Ge is extremely expensive for industrial application. Moreover, excessive Ge content makes the filler metal extremely brittle [22]. Previous research studies indicate that it is hard to balance the low melting temperature, high strength, satisfactory corrosion resistance, good forming performance, and low cost of the brazing filler metal during the application of NOCOLOK flux brazing technology.

In this study, a new type of Al-Si-Cu-Ni alloy was developed and made into brazing filler metal with melt-spinning technology. With the support of melt-spinning technology, the multiple alloyed brittle metal can not only be made into the foil shape, but also have high atomic activity, further decreasing the melting temperature of the alloy. Microstructure and thermal properties of the melt-spun ribbon was investigated. For potential application in the manufacturing of heat exchangers, brazing experiments of 3003 aluminum alloy with this melt-spun ribbon were carried out followed by the analysis of joint mechanical property, gas tightness, microstructure, and corrosion resistance performance.

## 2. Materials and Methods

**Figure 1:**
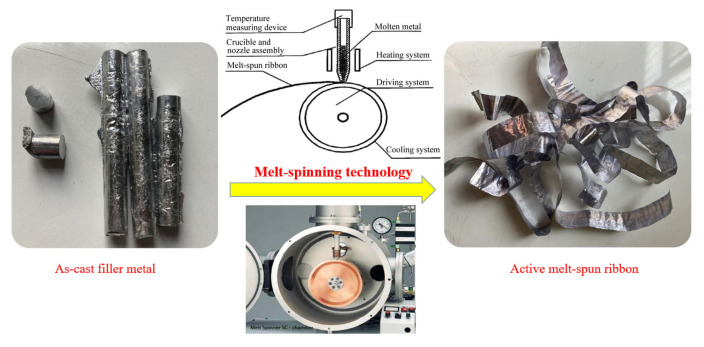
Schematic of melt-spun ribbon preparation process.

In the manufacturing of automotive heat exchangers, 3003 aluminum alloy is one of the most used materials. In this research, the commercialized 3003 aluminum alloy was utilized as the base material with dimensions of 10 × 20 × 2 mm^3^. The chemical composition of 3003 aluminum alloy is listed in Table 1. The solidus and liquidus temperatures of 3003 aluminum alloy are 643 °C and 655 °C, respectively. According to the alloying method developed by William Hume-Rothery [23,24,25], Al-Si-Cu-Ni alloy was applied in this research. The master alloys used for the preparation of Al-Si-Cu-Ni alloy were pure metals with the purity of 99.99%, including aluminum, silicon, copper, and nickel, respectively. Prior to this research, a series of Al-Si-Cu-Ni alloys were prepared by a vacuum induction melting furnace followed by a melting point test. The preliminary experiments revealed that the alloy with chemical composition of Al-5.0Si-20.5Cu-2.0Ni displayed a quite low melting temperature and smaller melting range. Consequently, Al-5.0Si-20.5Cu-2.0Ni alloy was selected as the low-melting-point filler metal in this research. In order to further reduce the melting point of brazing filler metal, melt-spinning technology was applied in this research. Figure 1 displays the schematic of melt-spinning technology. The melt-spinning process was carried out in a vacuum environment with a vacuum degree of 2.0 × 10^−2^ Pa. In order to obtain defect-free and micro-nano sized foil-like brazing filler metal, the rotational speed of the copper roller was set to be 1400 r/min, and the argon injection pressure in a quartz glass tube was set to be 0.02 MPa. During manufacturing, liquid metal was sprayed onto the rotating copper roller and solidified in a very short time. The thickness and width of the melt-spun ribbon was 0.1 mm and 20 mm, as shown in Figure 1. Due to the significant cooling capacity from the copper roller with high-speed rotation, the prepared melt-spun ribbon is characterized by higher atomic activity and lower melting temperature compared with the filler metal in as-cast condition. The conventional brazing flux containing K_3_AlF_6,_ which is widely used in NOCOLOK technology, is not applicable any more in this research due to its high melting point of 558 °C. In experiments, brazing flux CsAlF_4_ with lower melting point of 423–436 °C was employed to match that low-melting-point filler metal.

**Figure 2:**
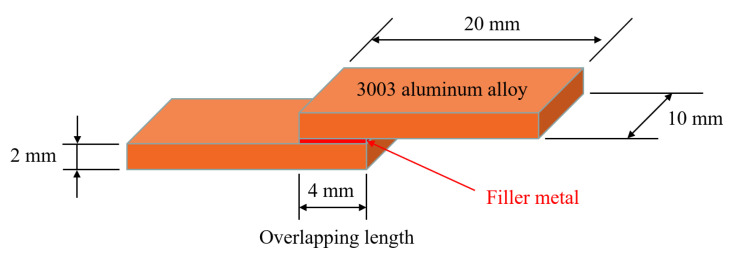
Schematic illustration of brazed specimens.

Before controlled atmosphere brazing, 3003 aluminum plates were cleaned in a NaOH solution with concentration of 10% to remove aluminum oxide film. Subsequently, the specimens were passivated in a HNO_3_ solution with concentration of 15%. After that, the specimens were cleaned in deionized water and dried waiting for brazing. The atmosphere during the brazing process was controlled by high purity N_2_, in which the oxygen concentration and dew-point temperature was controlled below 100 ppm and −40 °C, respectively. The thermal property of low-melting-point filler metal was determined by differential scanning calorimetry (DSC, Q100, TA Instruments, New Castle, DE, USA) and differential thermal analysis (DTA, STA449F3-QMS403D, Netzdsch, Selb, Germany), which was heated from room temperature to 600 °C with a heating rate of 10 °C/min under nitrogen protection. The microstructure of the filler metal and joint was observed with an optical microscopy and scanning electron microscopy (SEM, Carl Zeiss NTS GmbH, Merlin Compact, Jena, Germany) coupled with energy dispersive spectrometry (EDS). To evaluate corrosion resistance of the filler metal, the open circuit potential and corrosion current density of the filler metal as well as base material 3003 aluminum alloy were tested on an electrochemical workstation (Parstat 2273, Advanced Electrochemical System, Princeton Applied Research, Oak Ridge, TN, USA). For evaluation of the joint mechanical property, 3003 aluminum plates were lapped with an overlap length of 4 mm. Figure 2 shows the geometry and dimensions of the brazing specimen used for mechanical testing and microstructure observation. The joint shear strength was evaluated by an electronic universal testing machine (CMT5205, MTS Systems (China) Co. Ltd., Shenzhen, China) with the constant shear rate of 2 mm/min. For each brazing condition, five specimens were tested, and the average shear strength value was calculated to avoid experimental error.

**Figure 3:**
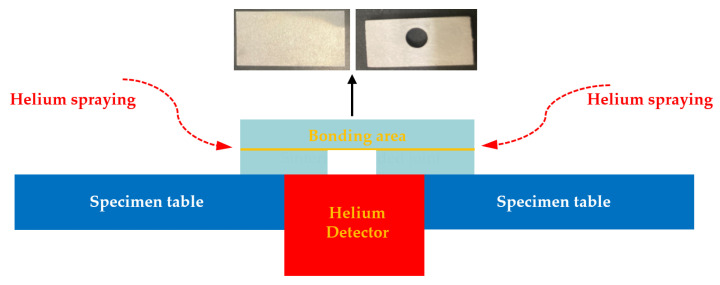
Schematic of gas tightness test for CAB 3003 aluminum joint.

In this research, a gas tightness test was carried out after CAB experiments to measure leakage rate of the joint. Gas tightness of brazed joints were tested with helium leak mass spectrometer ZQJ-530 (KYKY Technology Development Ltd., Beijing, China). Helium mass spectrum detection technology is characterized by high sensitivity and low effective minimum detectable leak rate, which is around 5 × 10^−12^ Pa·m^3^/s [26,27]. Figure 3 shows the schematic of helium mass spectrum detection technology. To measure gas tightness, a round through-hole was machined on one piece of 3003 aluminum alloy before the brazing process. After the CAB experiment, the specimen was put on the specimen table to measure gas tightness. The contact region between 3003 aluminum alloy and the specimen table was sealed by a sealant. Therefore, the bonding area was the only leakage path for helium into the detector.

## 3. Results

### 3.1. Melting Characteristics and Microstructure of Low-Melting-Point Filler Metal

**Figure 4:**
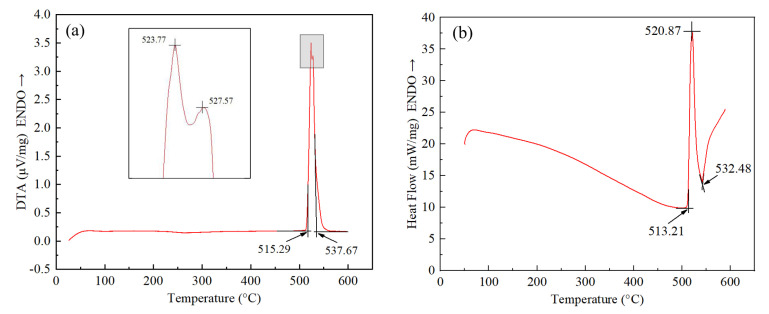
DTA and DSC curve of Al-5.0Si-20.5Cu-2.0Ni alloy in different condition: (**a**) As-cast condition; (**b**) Melt-spun ribbon.

The melting characteristics of filler metal are the basis to determine process parameters, such as brazing temperature. Figure 4 shows the DTA and DSC curve of Al-5.0Si-20.5Cu-2.0Ni alloy in different conditions. In Figure 4a, it can be seen that the solidus and liquidus of Al-5.0Si-20.5Cu-2.0Ni alloy in as-cast condition is 515.29 °C and 537.67 °C, whose liquidus is about 40 °C lower than that of the eutectic alloy of Al-12.6Si (577 °C). In the marked area, two endothermic peaks of 523.77 °C and 527.57 °C were observed, which is associated with the melting of Al-Si-Cu eutectic and Al-Cu eutectic formed in the casting process. Figure 4b shows the solidus and liquidus of filler metal made by melt-spinning technology is 513.21 °C and 532.48 °C, respectively. The solidus and liquidus of Al-5.0Si-20.5Cu-2.0Ni alloy decreased approximately 2 °C and 5 °C compared with the alloy in as-cast condition. Moreover, only one endothermic peak at 520.87 °C can be found in Figure 4b. The melting point test revealed that melt-spun ribbon had a lower melting point and narrower melting range compared to the same alloy in as-cast condition.

**Figure 5:**
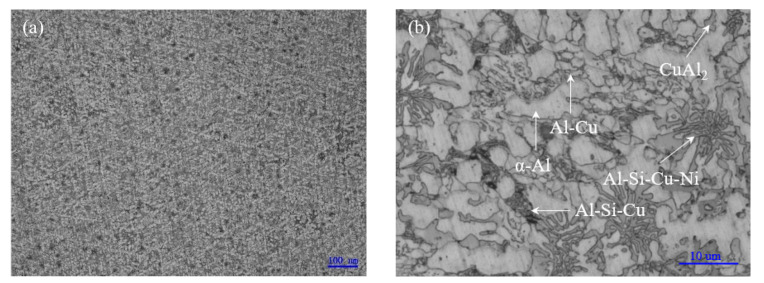
Optical microstructure of Al-5.0Si-20.5Cu-2.0Ni alloy in as-cast condition: (**a**) Magnification of 100×; (**b**) Magnification of 1000×.

As shown in Figure 5, it is believed that Al-5.0Si-20.5Cu-2.0Ni alloy in as-cast condition mainly contained the dendrite α-Al solid solution, CuAl_2_ (θ) intermetallic compound (IMC), Al-Cu binary phases in eutectic, Al-Si-Cu ternary phases in eutectic, and Al-Si-Cu-Ni quaternary phases in eutectic. Primary Si particle and Al-Si eutectic were not observed because of the low content of silicon. As is known, nickel and copper have a similar crystal structure making them have infinite solid solubility in both a liquid and solid state. With the addition of 2.0% nickel into the alloy, the continuously distributed IMC disappeared, replaced by Al-Si-Cu and Al-Si-Cu-Ni eutectic phase compared to Al-Cu eutectic alloy. Obviously, element segregation occurred in casting Al-5.0Si-20.5Cu-2.0Ni alloy, which made the corresponding DTA curve display multiple endothermic peaks.

**Figure 6:**
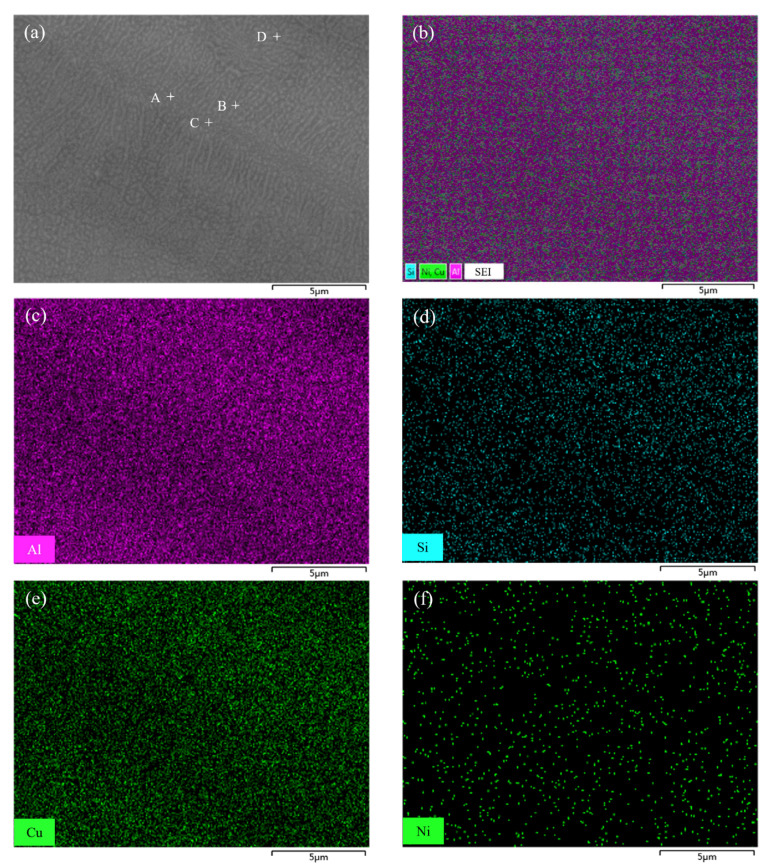
SEM micrograph of filler metal Al-5.0Si-20.5Cu-2.0Ni fabricated by melt-spinning technology: (**a**) SEM image; (**b**) Corresponding elemental mapping; (**c**–**f**) Individual elemental mapping of Al, Si, Cu, and Ni, respectively.

To analyze the elemental distribution of filler metal fabricated by melt-spinning technology, an EDS mapping was performed on the microstructure of Al-5.0Si-20.5Cu-2.0Ni ribbon. Figure 6a shows that the microstructure of filler metal is quite uniform and small, even the grain size is less than 500 nm. Compared with casting alloy, the melt-spun ribbon has finer microstructures and grain size, which enhances the atomic activity causing diffusion much more easily during the brazing process. In addition, the melting point of filler metal decreases with the formation of nanometer microstructural constituents. Figure 6b–f show the corresponding and individual EDS elemental mapping of melt-spun ribbon. Elements of Al, Si, Cu, and Ni in filler metal are evenly distributed and elemental segregation was not found. During the melt-spinning process, liquid metal will be quickly frozen at a cooling rate of 10^5^~10^6^ °C/s, which is much higher than that of traditional castings [28]. The changes in melting point and endothermic peak numbers of Al-5.0Si-20.5Cu-2.0Ni alloy primarily results from the microstructure evolution in different conditions. EDS analysis of 4 points marked in Figure 6a were carried out and results are summarized in Table 2. As can be seen, the chemical compositions of every testing point were quite close to the matrix alloy of Al-5.0Si-20.5Cu-2.0Ni, which meant that no intermetallic compounds and elemental segregation were formed.

### 3.2. Microstructure and Element Distribution Analysis of Brazed Joint

**Figure 7:**
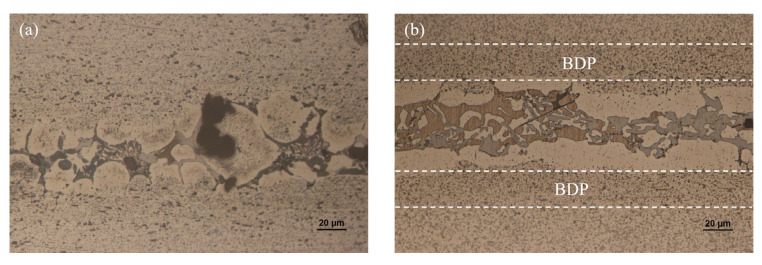

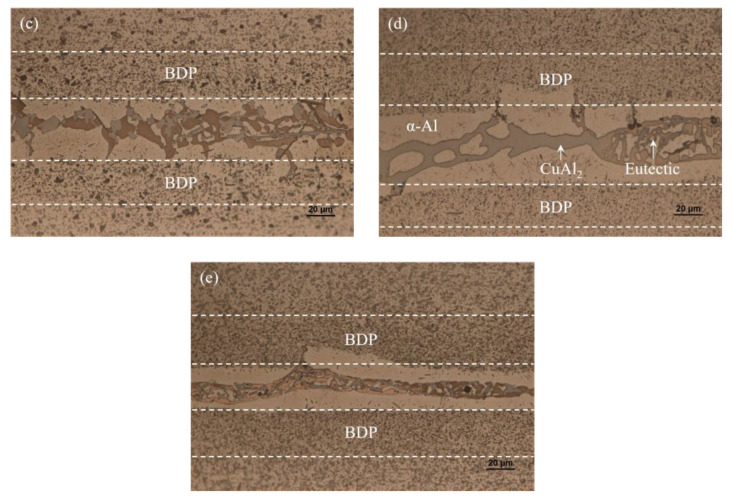
Microstructure of the joint brazed with different temperature and dwell time: (**a**) 565 °C and 15 min; (**b**) 570 °C and 15 min; (**c**) 575 °C and 15 min; (**d**) 575 °C and 20 min; (**e**) 575 °C and 25 min.

Figure 7 shows the image of the metallographic section of the joint after the CAB experiment. The microstructure was revealed by etching with Keller’s reactant. Re-solidification of the brazed joint shows up coarse α-Al crystal grains and multiphase deposits in light grey and brown contrast. Compared with melt-spun ribbon, the microstructure of the brazing seam was nonuniform anymore due to the slow cooling rate after the brazing process. As can be seen, the α-Al solid solution is mainly located on the bonding interface of 3003 and the multiphase deposits including low temperature eutectic phases; the intermetallic compounds are mainly located at the center of the brazing seam. The main reason is that α-Al solid solution and 3003 aluminum alloy substrates have the same crystal structure, leading to a lower nucleation energy. Consequently, α-Al crystal grains will grow on the surface of 3003 aluminum alloy. The remaining alloy elements left after diffusion are in the middle of the brazing seam in the form of eutectic or intermetallic compounds. The shape of eutectic and intermetallic compounds changes with brazing temperature and dwell time. As shown in Figure 7a, when the brazing temperature was set to be 565 °C, some voids appeared in the center of the brazing seam due to the poor fluidity of filler metal at a low temperature. As the brazing temperature increases, the fluidity of filler metal increases. Figure 7b shows that voids in the joint were greatly reduced. In the center of the brazing seam, the mixture of intermetallic compounds and eutectic phases were founded in the shape of net-like and blocky structures. As the brazing temperature reached 570 °C or higher, the diffusion of alloying elements from the liquid into base 3003 aluminum alloy led to solid-state precipitation in the so-called “band of dense precipitates” (BDP), as marked in Figure 7b–e. When the brazing temperature reached 575 °C, the void disappeared completely, as shown in Figure 7c–e. With the extension of dwell time from 15 min to 25 min, more alloying elements diffused into the base 3003 aluminum alloy. Therefore, the number of eutectic phases and intermetallic compounds, which were mainly composed of Cu, Si, Ni, and base Al, was gradually decreasing. Meanwhile, the eutectic phases and intermetallic compounds gradually accumulated to the center of the brazing seam. As shown in Figure 7e, when the dwell time was 25 min, the eutectic phases and intermetallic compounds even showed the characteristics of layered distribution, which was harmful to the mechanical properties of the joint.

**Figure 8:**
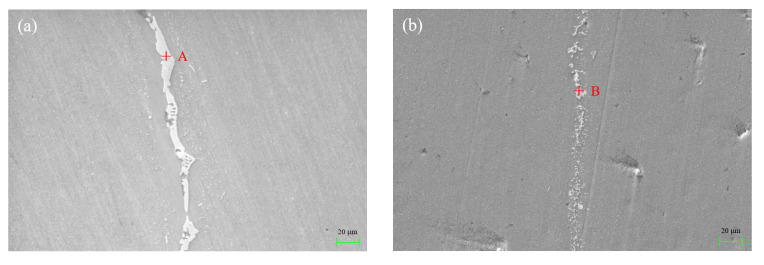

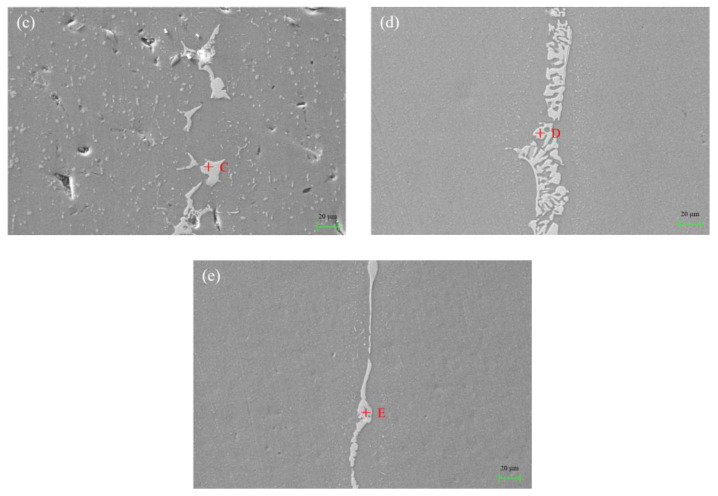
SEM image of the joint brazed with different temperature and dwell time: (**a**) 565 °C and 15 min; (**b**) 570 °C and 15 min; (**c**) 575 °C and 15 min; (**d**) 575 °C and 20 min; (**e**) 575 °C and 25 min.

Figure 8 shows the SEM image of the joint interface brazed with different temperatures and dwell times. As can be seen, a good metallurgical bond between filler metal and 3003 aluminum alloy can be obtained under experimental brazing conditions. Cu and Ni elements with large atomic numbers were mainly enriched in the bright area. The morphology of the bright area in the center of the brazing seam varied with the change of brazing conditions. As brazing temperature increased from 565 °C to 575 °C, the morphology of the bright area in the brazing seam changed from a typical coarse layer structure to a fine particle and large blocky structure gradually, as shown in Figure 8a–c. With the extension of dwell time from 15 min to 25 min, the morphology of the bright area changed from a large blocky structure to a net-like and uneven thin layer structure, as shown in Figure 8c–e. This indicated that the microstructure of the brazing seam was sensitive to the brazing temperature and dwell time. The appearance of the net-like microstructure was more beneficial to improve the mechanical properties of joints because there were more bonding interfaces between the bright area and matrix [29]. Table 3 displays the chemical compositions (at.%) of points marked in Figure 8. It can be summarized that the atomic ratio of Al to Cu and Ni at point A to point D was close to 2:1. It can be deduced that these bright phases were intermetallic compound CuAl_2_ with a slight replacement between the atom of copper and nickel. In point E, all elements including Al, Si, Cu, and Ni in filler metal can be found. This indicated that this area mainly contained eutectic phases and intermetallic compounds.

**Figure 9:**
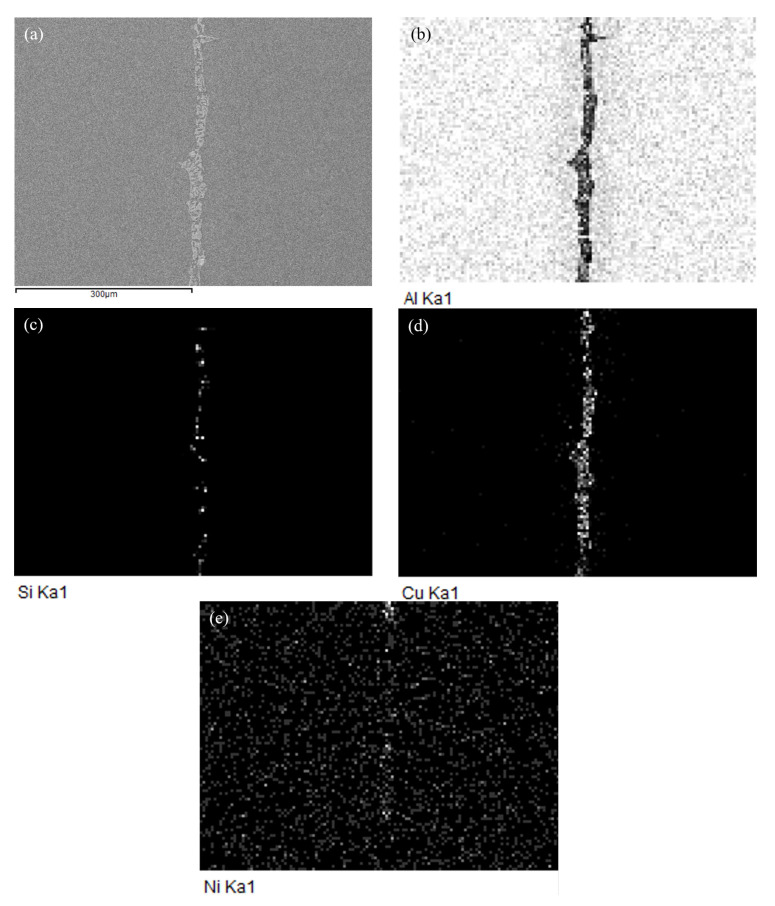
SEM micrograph at the interface of 3003 joint brazed at 575 °C for 20 min, and energy dispersive X-ray maps showing distribution of elements: (**a**) SEM image; (**b**–**e**) individual elemental mapping of Al, Si, Cu and Ni, respectively.

Figure 9 shows SEM micrograph at the interface of the 3003 joint brazed at 575 °C for 20 min, and the corresponding energy dispersive X-ray maps show distribution of elements Al, Si, Cu, and Ni. Compared with the microstructure of filler metal shown in Figure 6, the energy dispersive X-ray maps indicated that the composition of filler metal greatly changed during the brazing reaction with substrate of 3003 aluminum alloy. The elemental mapping in Figure 9 revealed that Al content in the joint decreased significantly after brazing. The depletion of Al was replaced by the enrichment of Cu and a small amount of Si. The content of Ni in the brazing seam was very small, which was mainly dependent on the distribution of Cu in the center of the brazing seam. As can be seen, most of the melting reducing elements including Cu and Si remained in the brazing seam due to a lower brazing temperature and dwell time. This indicated that it was not easy for dissolution defects to occur below the brazing temperature of 575 °C.

### 3.3. Gas Tightness and Mechanical Property of Brazed Joint

Heat exchangers require strict sealing performance to protect coolant from leakage. For CAB components, the brazing area is the only path for leakage. The brazing defects, e.g., incomplete brazing area, pore, and so on, were the primary leakage reason. Table 4 shows the testing results of joint gas tightness. Five specimens were measured for each brazing condition and the maximum leak rate was displayed. As can be seen in Table 4, the leak rate after brazing was qualified to be 10^−10^ Pa·m^3^/s when the brazing temperature was 570 °C or higher. When the brazing temperature was 565 °C, the leak rate was 10^−8^ Pa·m^3^/s. The gas tightness test was performed on the same specimens after one week, and results showed that no changes can be detected on these specimens. Though some micro-voids were found in the joint microstructure, gas tightness of the joints brazed with temperature of 570 °C or higher were qualified, whether the gas tightness test was carried out immediately after brazing or after one week. This indicated that the micro-voids existing in the joint were disconnected in the brazing area. 

**Figure 10:**
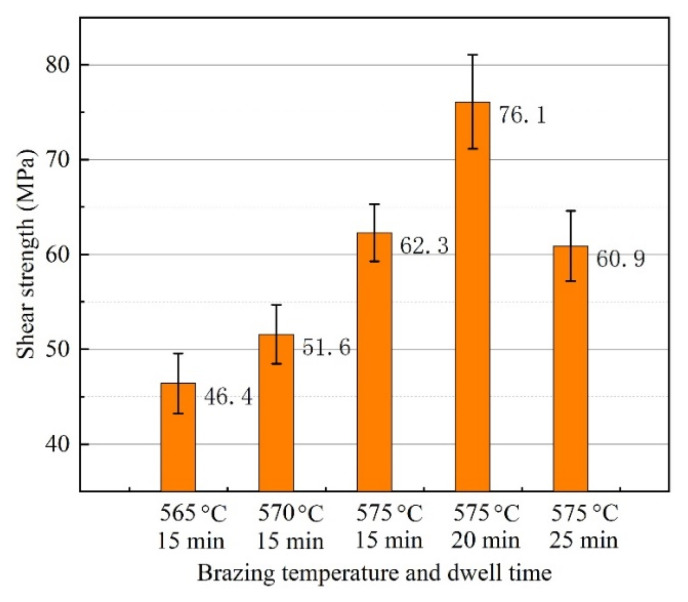
Shear strength of the joint brazed at different temperature and dwell time.

Shear strength of 3003 joints brazed with various temperatures and dwell times is demonstrated in Figure 10. Since the purpose of this research is to develop a low-melting-point brazing filler metal for the bonding of 3003 aluminum alloy, the maximum brazing temperature was set to be lower than the eutectic point of traditional Al-12.6Si alloy. When the brazing condition was set to be 565 °C with 15 min, the minimum joint shear strength of 46.4 MPa was obtained. Since the brazing temperature of 565 °C was so low, a good joint of 3003 aluminum joining with filler metal Al-5.0Si-20.5Cu-2.0Ni could not be formed. That was revealed in the microstructure analysis and gas tightness test as well. As the brazing temperature increased from 565–575 °C, the bonding strength improved from 46.4–62.3 MPa. Microstructure images showed that there were little parts of the surface left unbonded when the brazing temperature reached 570 °C or higher. With brazing temperature 575 °C, the satisfactory bonding strength increased to 76.1 MPa when the dwell time was extended to 20 min. When the dwell time was extended to 25 min, the shear strength reduced to 60.9 MPa. This indicated that more dwell time may induce growth of the interfacial layer, which can be confirmed from the evolution of the joint microstructure, and consequently reduce the joint shear strength. 

### 3.4. Open Circuit Potential and Corrosion Current Density of Filler Metal and Base Metal

**Figure 11:**
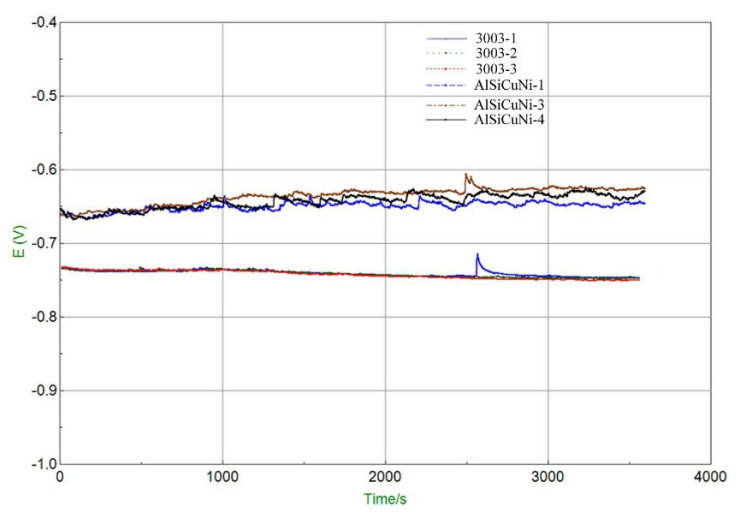
Open circuit potential fitting curve of filler metal and base metal.

The open-circuit potential (OCP) fitting curve refers to the curve of potential variation with time between the working electrode and reference electrode when the current density is zero. It can be used to detect the natural corrosion potential to judge the corrosion tendency of material. The higher the natural corrosion potential is the less likely the sample is to be corroded. In addition to this, OCP experiments can also provide stable test conditions for subsequent polarization experiments. Figure 11 shows OCP fitting curves of filler metal and 3003 aluminum alloy. Three specimens were tested for each type of material. As can be seen, the potential of filler metal increases to a certain extent at the initial stage of corrosion and then tends to be stable gradually, while the potential of 3003 aluminum remains relatively stable throughout. The OCP value at testing time of 3000 s, 3300 s, and 3600 s are listed in Table 5. The average OCP of filler metal and 3003 aluminum alloy was −627 mV and −746 mV, respectively. Obviously, the OCP value of filler metal was 119 mV higher than that of 3003 aluminum alloy. This indicates that filler metal is not easily corroded and its corrosion resistance is better than that of 3003 aluminum alloy.

**Figure 12:**
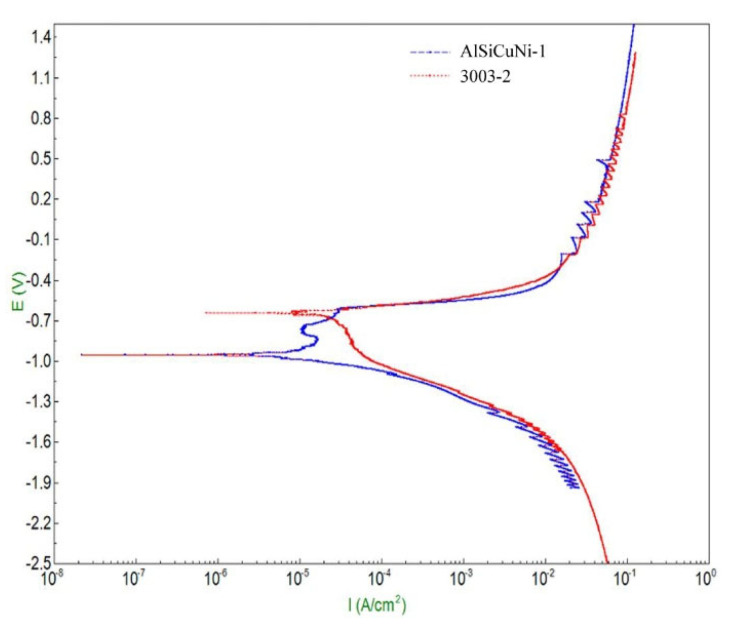
Polarization potential test of filler metal and base metal.

Tafel curve extrapolation method was employed to obtain the alloy’s corrosion current density Icorr in this research, as shown in Figure 12. The corrosion current density Icorr reflects the electron transfer rate, representing the corrosion rate of the alloy. The smaller the corrosion current density is, the smaller the corrosion rate is, which means that the alloy has a better corrosion resistance. As summarized in Table 6, Icorr of filler metal and 3003 aluminum alloy was 1.3276 × 10^−4^ A/cm^2^ and 2.3605 × 10^−4^ A/cm^2^, respectively. It can be found that Icorr of filler metal was 43.8% lower than that of 3003 aluminum alloy. Based on the test results of the OCP and corrosion current density, it can be concluded that the brazing filler metal had better corrosion resistance than that of 3003 aluminum alloy. During the service of heat exchangers, better corrosion resistance of filler metal can make the brazing joint stable, which makes it have longer service life.

## 4. Conclusions

In this research, a new low-melting-point brazing filler metal Al-5.0Si-20.5Cu-2.0Ni was prepared by using melt-spinning technology and then was applied to the CAB of 3003 aluminum alloy. The melting characteristics and microstructure of filler metal was researched and the microstructure, element distribution, gas tightness, and shear strength of the joint were studied. The OCP and corrosion current density of filler metal and 3003 aluminum alloy were tested. The conclusions can be summarized as follows:

(1) The solidus and liquidus of brazing filler metal Al-5.0Si-20.5Cu-2.0Ni made by melt-spinning technology was 513.21 °C and 532.48 °C, respectively. Elements of Al, Si, Cu, and Ni in melt-spun ribbon were evenly distributed and elemental segregation was not found. The microstructure of brazing filler metal was uniform and small.
(2) When the brazing temperature was 565 °C, some voids appeared in the center of the brazing seam. As the brazing temperature reached 575 °C, the void in the joint disappeared completely. The morphology of CuAl_2_ was sensitive to the brazing temperature and dwell time. The appearance of net-like CuAl_2_ brazed at 575 °C for 20 min was more beneficial to improve joint mechanical properties.
(3) The leakage rate of the joint was qualified to be 10^−10^ Pa·m^3^/s when the brazing temperature was 570 °C or higher, whether the gas tightness test was carried out immediately after brazing or after one week. The maximum shear strength of 76.1 MPa can be obtained when the joint was brazed at 575 °C for 20 min. More dwell time induced the growth of the interfacial layer and reduced the joint shear strength.
(4) The brazing filler metal Al-5.0Si-20.5Cu-2.0Ni had better corrosion resistance than that of 3003 aluminum alloy. The average OCP of filler metal and 3003 aluminum alloy was −627 mV and −746 mV. The Icorr of filler metal was 43.8% lower than that of 3003 aluminum alloy.

## Figures and Tables

**Table 1:** Chemical composition of 3003 aluminum alloy used in heat exchanger (in wt.%).

Element	Si	Fe	Cu	Mn	Al
wt.%	0.12	0.70	0.12	1.20	Balance

**Table 2:** EDS results (wt.%) of points marked in Figure 6.

Test Points	Al	Si	Cu	Ni
A	73.7	4.5	19.9	1.9
B	71.6	4.9	21.4	2.1
C	72.1	5.1	20.9	1.9
D	74.3	4.8	18.8	2.1

**Table 3:** Chemical compositions (at.%) of points marked in Figure 8.

Test Points	Al	Si	Cu	Ni	C
A	57.19	-	20.81	12.70	9.30
B	63.92	-	16.07	9.10	10.91
C	55.79	-	24.02	8.80	11.39
D	59.01	0.96	25.37	3.23	11.43
E	62.37	5.74	13.10	8.77	10.02

**Table 4:** Gas tightness of 3003 aluminum joint brazed with melt-spun ribbon at different brazing conditions.

CAB Temperature (°C)	Dwell Time (min)	Leak Rate after Brazing (Pa·m^3^/s)	Leak Rate after One Week (Pa·m^3^/s)
565	15	10^−8^	10^−8^
570	15	10^−10^	10^−10^
575	15	10^−10^	10^−10^
575	20	10^−10^	10^−10^
575	25	10^−10^	10^−10^

**Table 5:** Open circuit potential of filler metal and base metal at different time.

Specimen No.	OCP (mV)			Average OCP (mV)
	3000 s	3300 s	3600 s	
AlSiCuNi-1	−618	−619	−619	−627
AlSiCuNi-2	−625	−624	−620	
AlSiCuNi-3	−638	−642	−640	
3003-1	−745	−747	−747	−746
3003-2	−747	−748	−747	
3003-3	−745	−747	−747	

**Table 6:** Polarization curve fitting results of filler metal and base metal.

Specimen	Icorr (A/cm^2^)
AlSiCuNi	1.3276 × 10^−^^4^
3003	2.3605 × 10^−^^4^
